# Supplementary material for: Geologically constrained 2-million-year-long simulations of Antarctic Ice Sheet retreat and expansion through the Pliocene
Source: Nat Commun. 2024 Aug 15;15:7014. doi: 10.1038/s41467-024-51205-z (PMC11327337; doi:10.1038/s41467-024-51205-z)
Supplement: Supplementary file 1 — Supplementary Information [file 41467_2024_51205_MOESM1_ESM.pdf]

Supplemental Figures

Geologically constrained 2-million-year-long simulations of Antarctic Ice Sheet retreat and expansion through the Pliocene

Anna Ruth W. Halberstadt*[1], Edward Gasson[2], David Pollard[3], James Marschalek[4], Robert M. DeConto[5]

[1] Department of Earth and Planetary Sciences, Jackson School of Geosciences, The University of Texas at Austin, Austin, TX, USA
[2] School of Geographical Sciences, University of Bristol, Bristol, UK
[3] Earth and Environmental Systems Institute, Pennsylvania State University, University Park, PA, USA
[4] Department of Earth Science and Engineering, Imperial College London, London, UK
[5] Department of Geosciences, University of Massachusetts Amherst, Amherst, MA, USA

** arhalberstadt@utexas.edu*

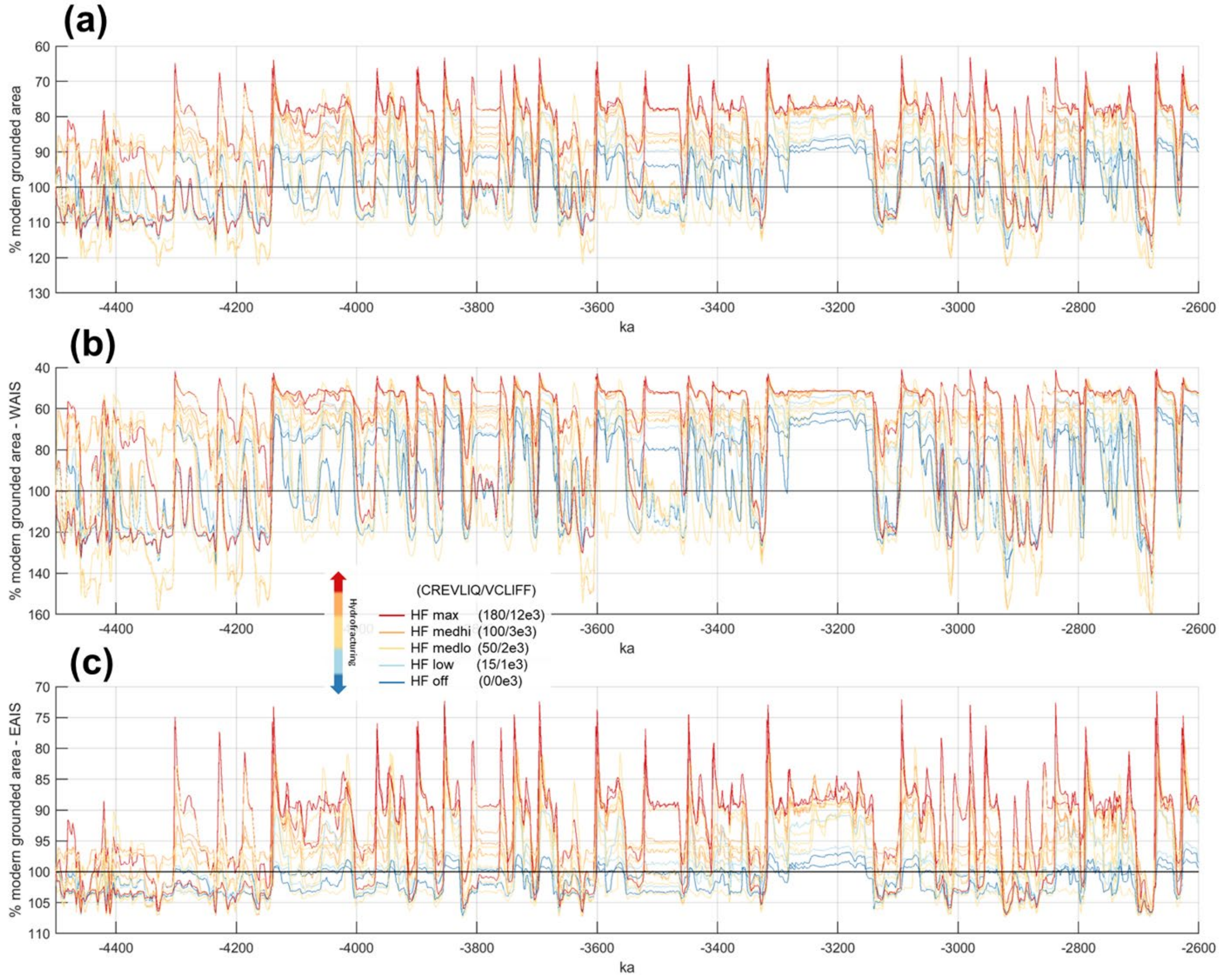

***Figure S1**. Model ensemble runs, colored by hydrofracturing parameter combinations (see text for parameter description), are plotted with respect to grounded ice area change compared to modern for (a) the entire ice sheet, (b) West Antarctic Ice Sheet (WAIS) only, and (c) East Antarctic Ice Sheet (EAIS) only.*

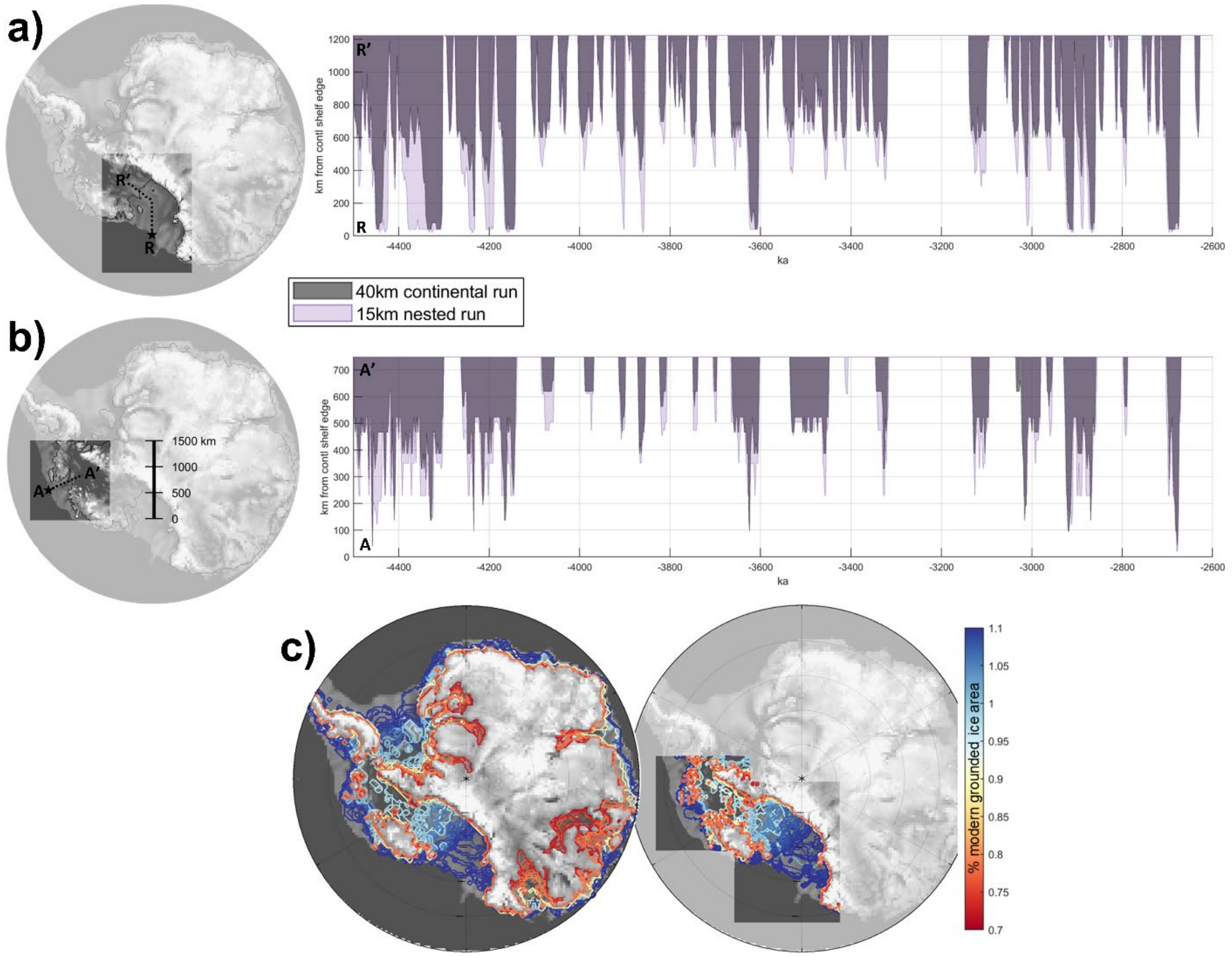

***Figure S2. Insensitivity of results to model resolution**. Higher-resolution (15km) nested simulations, driven by boundary conditions from a best-fit continental run (40km resolution), produce very similar grounding-line fluctuation patterns (a,b) and extents (c). (a,b) Grounding-line behavior at each timestep is plotted with respect to distance from the continental shelf edge (black star) along the trackline shown in the left-hand-side plot (dashed black line) in the (a) Ross Sea and (b) Amundsen Sea. (c) Nested simulations produce the same grounding line positions through time (right) as the continental simulation (left). Grounding line color indicates the ice sheet grounded area with respect to the modern extent.*

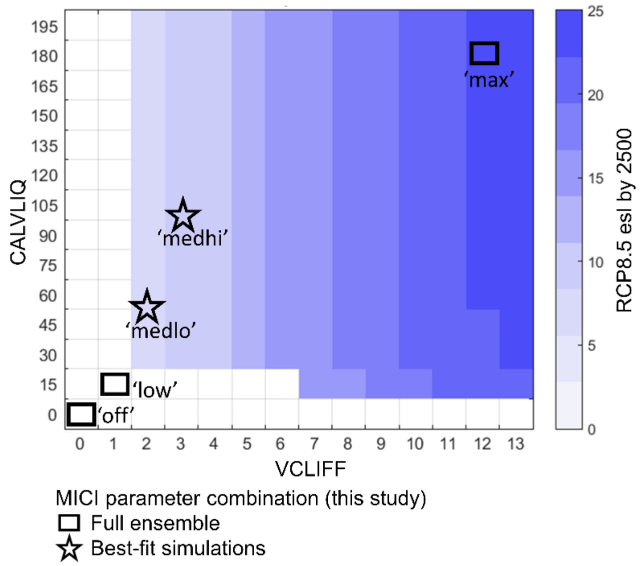

Figure S3: Marine Ice Cliff Instability (MICI) parameter combinations from this study compared to DeConto et al.*[1] *future sea level projections. *MICI parameter combinations from DeConto et al. (2021) that passed the Pliocene sea level constraint (11-21 m esl); color scheme shows long-term future sea level projections (RCP8.5, by the year 2500). In this study, the two best-fit simulations have intermediate MICI parameterizations ('medhi' and 'medlo'; also see Fig. 1c), which fall into the lower end of the DeConto et al. (2021) range.*

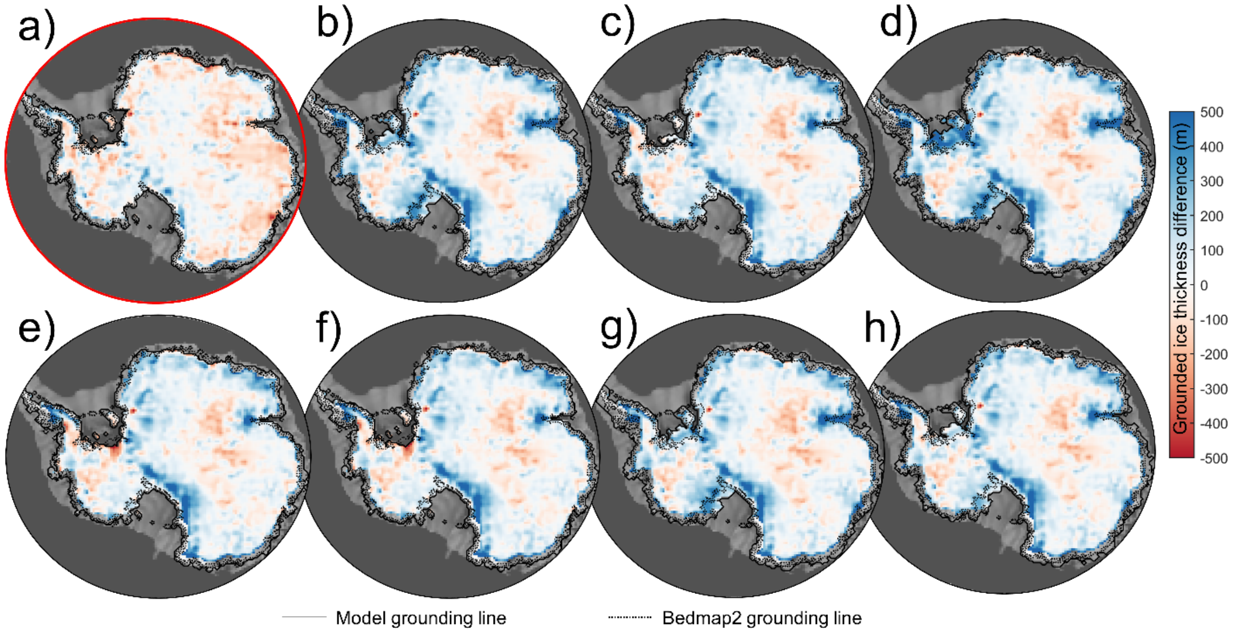

Figure S4. Model simulations under constant preindustrial matrix-interpolated climate*. Each parameter combination in our ensemble is tested under preindustrial conditions. Simulations are run for 2000 years with constant modern orbital parameters and CO_2 concentrations set at 280 ppm, and the resulting ice thickness and grounding line is compared to Bedmap2[2]. Only a subset of model parameter combinations is shown here; (a) Initial conditions; (b) OC3-HFlo-area; (c) OC3-HFoff-vol; (d) OC3-HFmedlo-area; (e) OC4-HFmedlo-vol; (f) OC4-HFmedhi-vol; (g) OC3-HFmax-area; (h) OC3-HFmedhi-vol.*

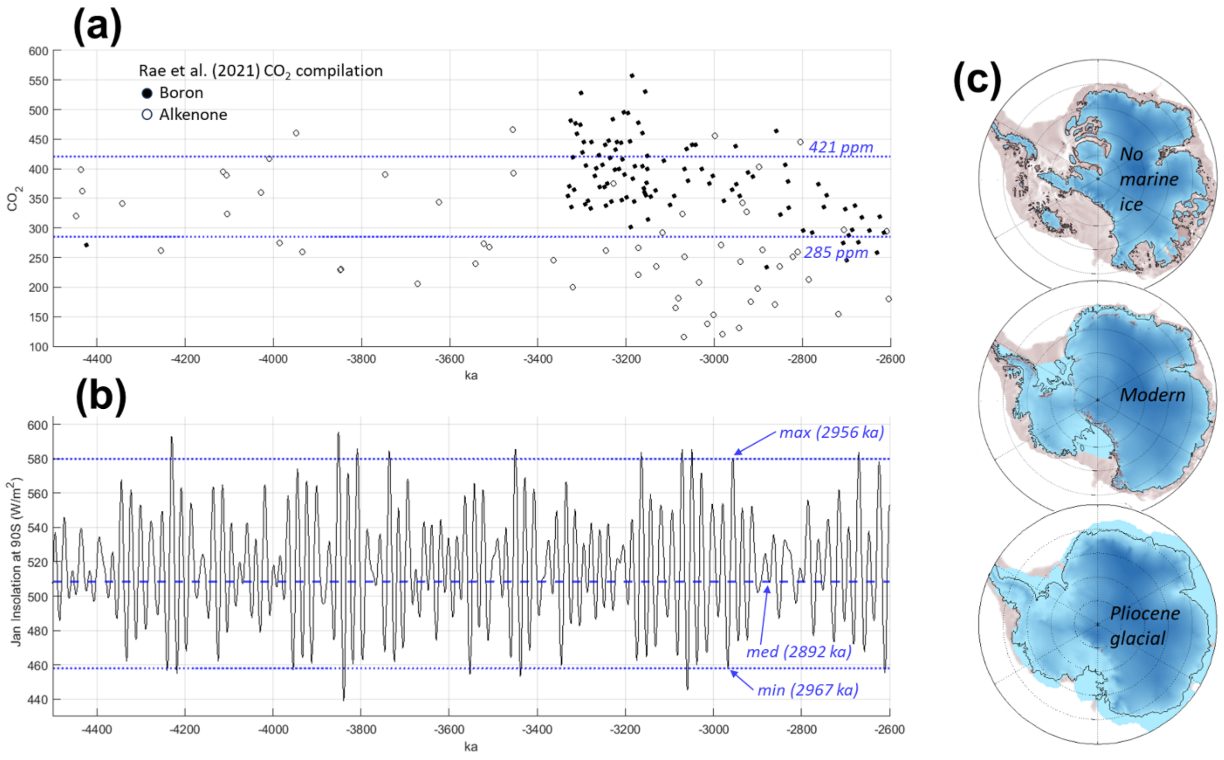

Figure S5: End-member parameters for the climate matrix snapshots. *(a) CO_2 end-member values were chosen based on the maximum and minimum CO_2 concentrations reconstructed from an orbital-resolution dataset (de la Vega et al.,[3]; across the KM5c interval). (b) Time periods characterized by maximum, minimum, and median Antarctic summer insolation values during the G17 interval provided obliquity, eccentricity, and precession values to construct 'maximum-insolation orbit', 'minimum-insolation orbit', and 'median-insolation orbit' matrix snapshots (respective values - obliquity: 23.80°, 22.71°, 22.89°; eccentricity: 0.0396, 0.0382, 0.0073; precession angle: 68.6°, 245.5°, 155.0°). (c) Surface topography is either 'no marine ice', with ice loss from marine-based portions of West and parts of East Antarctica (DeConto et al.,[1] Pliocene simulation); modern (Bedmap2[2]); or a Pliocene glacial configuration from a simulation forced by a climate model output with 285 ppm CO_2, a 'cold' astronomical orbit, and eustatic sea level at -60m.*

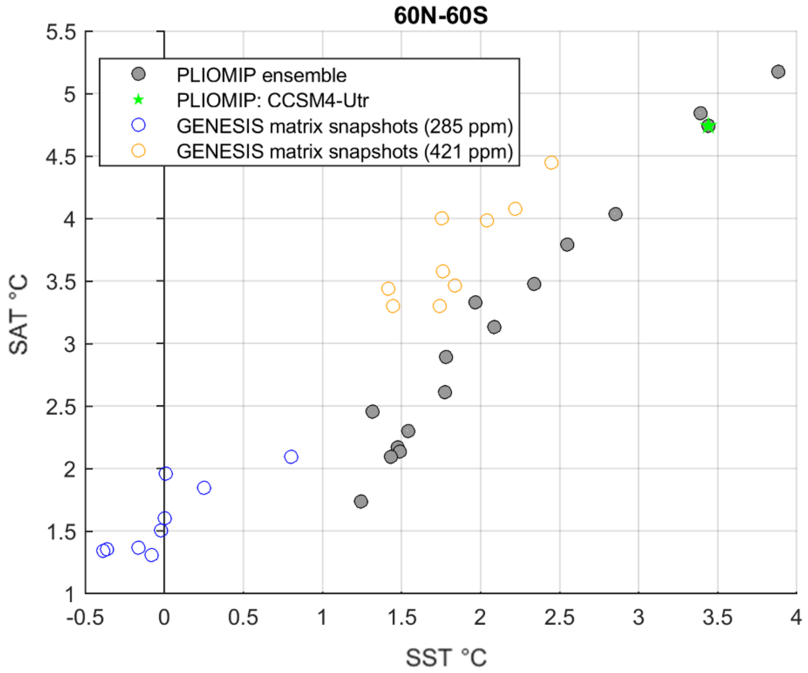

***Figure S6. Climate model snapshots compared to PlioMIP2 ensemble members**. Climate model snapshots using the GENESIS GCM (Global Climate Model) with 421 ppm CO_2 produce Pliocene climatologies (surface air temperatures and sea surface temperatures) that fall within the PlioMIP2 ensemble range of variability[4]. Note that the GENESIS GCM has a slab ocean model, while the PlioMIP2 simulations are fully coupled ocean-atmosphere models. Climate model snapshots with lower CO_2 expand below the PlioMIP2 range; PlioMIP2 simulations were conducted under 400 ppm CO_2 concentrations to reconstruct the KM5c interval (when orbital forcing was close to modern, though Pliocene warming was not peak), while we explore a wider range of glacial/interglacial conditions.*

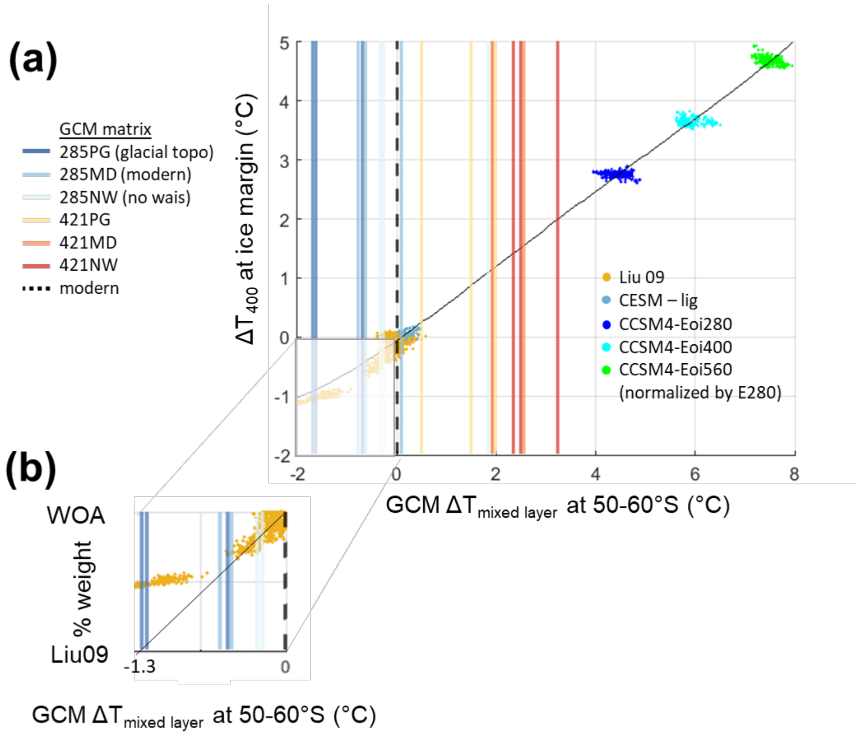

Figure S7: Ocean temperature scaling methodology. *Subsurface ocean temperatures that are used as input into the ice sheet model are calculated based on upper ocean (mixed-layer) temperature anomalies from the matrix of GCM (Global Climate Model) simulations. Mixed-layer ocean temperature anomalies (from 50-60°S latitudes only, to avoid differences due to ice sheet extent) are empirically related to subsurface ocean temperatures at the ice margin (the ice sheet model input quantity) by a quadratic relationship (black line). This empirical relationship is established based on comparing 50-60°S mixed-layer temperatures (GCM ΔT) to subsurface ice-margin temperatures (ΔT_{400}) in five fully coupled Earth Model simulations (Methods). For positive GCM ocean temperature anomalies (i.e., warmer ocean conditions than modern), subsurface ocean temperatures are supplied to the ice sheet model from on a modern distribution of 400-m-water-depth temperatures[5] with the addition of the empirically calculated subsurface temperature scaling (ΔT_{400}) at each time step. For negative GCM ocean temperature anomalies, i.e., colder ocean conditions than modern, subsurface ocean temperatures supplied to the ice sheet model are interpolated between a 'glacial' ocean ('Liu09')[6] and the modern climatology (World Ocean Atlas, 'WOA')[5] based on GCM mixed-layer temperatures at each time step (shown in inset).*

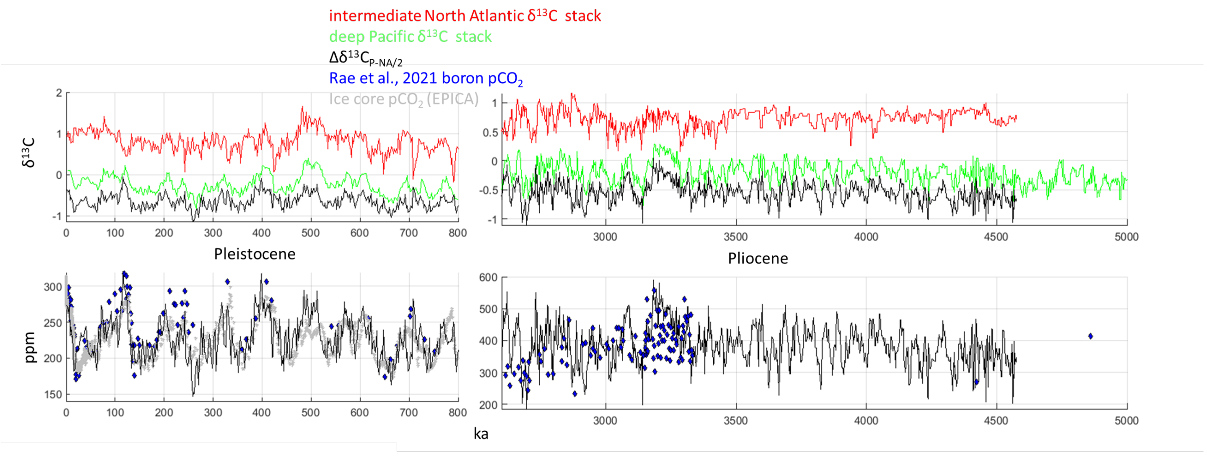

Figure S8: Orbital-resolution CO_2 dataset. *A continuous orbital-scale-resolution Pliocene CO_2 time series is constructed using a benthic $\delta^{13}C$-based proxy for atmospheric CO_2, following the approach of Lisiecki (2010)[7]. Individual $\delta^{13}C$ records from the deep Pacific and intermediate North Atlantic are stacked, and a combined $\Delta\delta^{13}C_{P\text{-}NA/2}$ curve is calculated and scaled to the mean and amplitude of Pliocene boron isotope-based CO_2 reconstructions[8]. Upper and lower left-hand plots demonstrate this methodology for the last 800 ka (see Lisiecki, 2010); right-hand plots illustrate the application of this approach using $\delta^{13}C$ Pliocene datasets to produce a CO_2 time series at sufficient temporal resolution to drive the ice sheet modeling efforts described here.*

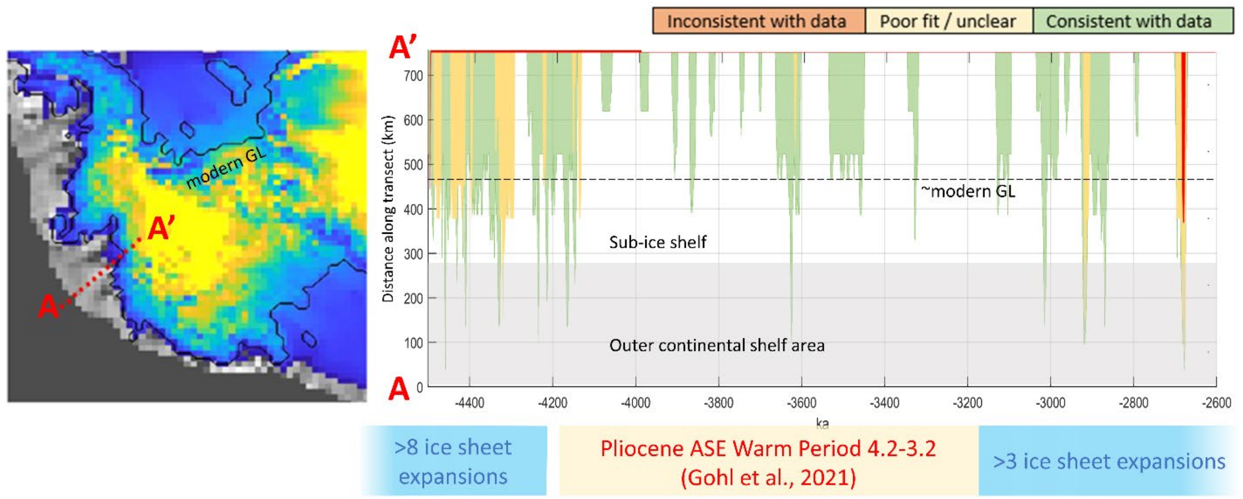

Figure S9: Model-data comparison example – grounding line behavior across the Amundsen Sea continental shelf. *In the Amundsen Sea sector, marine seismic and drill core data indicate a period of prolonged mid-Pliocene warmth, when the ice sheet was primarily receded, sandwiched by cooler periods in the earlier and later Pliocene characterized by multiple glacial advances across the continental shelf*[9,10]*. Ice sheet model simulations are compared to this geologic criterion by plotting modeled grounding line (GL) position across the continental shelf (along the red transect). This plot demonstrates the model-data evaluation process by highlighting three different simulations: the green model run is consistent with the geologic evidence because the grounding line advances across the outer continental shelf multiple times in the earlier and later Pliocene but is primarily receded during the identified 'Pliocene Amundsen Sea (ASE) Warm Period'. The yellow model run has a poorer fit to the geologic evidence because while the grounding line advances periodically across the inner continental shelf, it rarely expands beyond the modern configuration and therefore would not have been able to deposit the observed seismic signatures on the outer continental shelf. The red model run is inconsistent with the data because the grounding line in this region is mostly receded throughout the Pliocene, and never expands significantly beyond the modern configuration.*

Table S1: Geologic criteria for model evaluation

		Region	Geologic data type	Data-based criteria for model evaluation	Reference
Marine grounding line behavior	Ice sheet advance and retreat pattern	Amundsen Sea Embayment	Seismic surveys of continental shelf erosional unconformities; drill core sedimentation	≥8 early Pliocene grounding line advances across the continental shelf; mostly receded ice sheet from ~4.2-3.2 Ma; ≥3 late Pliocene grounding line advances across the shelf	[9,10] *(Gohl et al., 2021, Gille-Petzoldt et al., 2022)*
		Ross Sea Embayment	Seismic surveys and drill core record of continental shelf erosional unconformities	≥7-10 grounding line advances across the shelf; ≥13 grounding line advances across the ANDRILL-1B site after 3.4 Ma	[11–15] *(De Santis et al., 1995; Alonso et al., 1992; Bart et al., 2000; Naish et al., 2009; McKay et al., 2012)*
		Weddell Sea Embayment	Seismic surveys of continental shelf erosional unconformities and trough mouth fan formation	At least one grounding line advance to the shelf edge	[16–18] *(Bart et al., 1999; Smith & Anderson, 2010; Huang & Jokat 2016)*
		Wilkes Subglacial Basin	Drill core sediment interpretation; seismic surveys	≥12 grounding line advances to continental shelf break, punctuated by continental shelf retreat; periodic 'ice sheet collapse' events characterized by significant (~200km) inland grounding line retreat	[19–27] *(Orejola and Passchier, 2013; Reinardy et al., 2015; Cook et al., 2013; Patterson et al., 2014; Hansen et al., 2015; Bertram et al., 2018; Escutia et al., 2005; Williams et al 2010; Cook et al., 2014)*
	Extent of ice retreat	Wilkes Subglacial Basin	Ice rafted debris analysis; geochemical provenance	No grounding line retreat into the Adelie craton region during most interglacials	[28] *(Marschalek et al., 2022)*
		Aurora Subglacial Basin	Iceberg rafted debris analysis; seismic surveys and drill core record of continental shelf erosional unconformities	Large-scale grounding line retreat occurred periodically; grounding line retreat did not exceed 150km inland from the modern configuration	[26,27,29] *(Williams et al 2010; Cook et al., 2014; Gulick et al., 2017)*
		Prydz Bay	Seismic surveys of continental shelf erosional unconformities and trough mouth fan formation; drill core sediment	Periodic grounding line advance to outer shelf and grounding line retreat to approximately modern position with no ice shelf; at least one period of open marine sedimentation (no ice	[30–35] *(Cooper et al., 1991; O'Brien & Harris, 1996; Passchier et al, 2003; Passchier, 2011; Whitehead et al., 2004; Passchier & Whitehead, 2006)*

			interpretation; terrestrial outcrop sediments; iceberg rafted debris analysis	sheet or ice shelf) at inland outcrop locations	
Terrestrial records		ANDRILL catchment region (Wilkes Subglacial Basin)	Cosmogenic nuclide exposure ages of transported sediments	Spatially and temporally limited ice sheet retreat over the ANDRILL-1B catchment area; i.e., no large-scale Wilkes Subglacial Basin collapse	[36] *(Shakun et al., 2018)*
	Ice thicknesses	Interior nunataks	Cosmogenic nuclide exposure ages at nunataks	Ice sheet thickened by specified amounts at coastal and interior nunatak locations	[37] *(Yamane et al., 2015)*
		Pirrit Hills nunatak	Elevation transect of cosmogenic nuclide exposure ages	Long-term ice cover frequency pattern at the Pirrit Hills	[38,39] *(Spector et al., 2020; Halberstadt et al., 2023)*

1. DeConto, R. M. *et al.* The Paris Climate Agreement and future sea level rise from Antarctica. *Nature* **593**, (2021).

2. Fretwell, P. *et al.* Bedmap2: Improved ice bed, surface and thickness datasets for Antarctica. *Cryosphere* **7**, 375–393 (2013).

3. de la Vega, E., Chalk, T. B., Wilson, P. A., Bysani, R. P. & Foster, G. L. Atmospheric CO2 during the Mid-Piacenzian Warm Period and the M2 glaciation. *Sci. Rep.* **10**, 1–9 (2020).

4. Haywood, A. M. *et al.* The Pliocene Model Intercomparison Project Phase 2: Large-scale climate features and climate sensitivity. *Clim. Past* **16**, 2095–2123 (2020).

5. Levitus, S. *et al.* World ocean heat content and thermosteric sea level change (0-2000m), 1955-2010. *Geophys. Res. Lett.* **39**, 1–5 (2012).

6. Liu, Z. *et al.* Transient Simulation of Last Deglaciation with a New Mechanism for Bølling-Allerød Warming. *Science (80-.).* **325**, 310–314 (2009).

7. Lisiecki, L. E. A benthic δ13C-based proxy for atmospheric pCO2 over the last 1.5 Myr. *Geophysical Research Letters* vol. 37 (2010).

8. Rae, J. W. B. *et al.* Atmospheric CO2 over the past 66 million years from marine archives. *Annu. Rev. Earth Planet. Sci.* **49**, 609–641 (2021).

9. Gohl, K. *et al.* Evidence for a Highly Dynamic West Antarctic Ice Sheet During the Pliocene. *Geophys. Res. Lett.* **48**, 1–11 (2021).

10. Gille-Petzoldt, J. *et al.* West Antarctic Ice Sheet Dynamics in the Amundsen Sea Sector since the Late Miocene — Tying IODP Expedition 379 Results to Seismic Data. *Front. Earth Sci.* **10**, 1–19 (2022).

11. De Santis, L., Anderson, J. B., Brancolini, G. & Zayatz, I. Seismic Record of Late Oligocene Through Miocene Glaciation on the Central and Eastern Continental Shelf of the Ross Sea. *Geol. Seism. Stratigr. Antarct. Margin, Antarct. Res. Ser.* **68**, 235–260 (1995).

12. Alonso, B., Anderson, J. B., Diaz, J. I. & Bartek, L. R. Pliocene-Pleistocene Seismic Stratigraphy of the Ross Sea: Evidence for Multiple Ice Sheet Grounding Episodes. *Antarct. Res. Ser.* **57**, 93–103 (1992).

13. Bart, P. J., Anderson, J. B., Trincardi, F. & Shipp, S. S. Seismic data from the Northern basin, Ross Sea, record extreme expansions of the East Antarctic Ice Sheet during the late Neogene. *Mar. Geol.* **166**, 31–50 (2000).

14. Naish, T. R. *et al.* Obliquity-paced Pliocene West Antarctic ice sheet oscillations. *Nature* **458**, 322–328 (2009).

15. McKay, R. *et al.* Antarctic and Southern Ocean influences on Late Pliocene global cooling. *Proc. Natl. Acad. Sci.* **109**, 6423–6428 (2012).

16. Bart, P. J., De Batist, M. & Jokat, W. Interglacial collapse of crary trough-mouth fan, weddell sea, antarctica: implications for antarctic glacial history. *J. Sediment. Res.* **69**, 1276–1289 (1999).

17. Smith, R. T. & Anderson, J. B. Ice-sheet evolution in James Ross Basin, Weddell sea margin of the Antarctic peninsula: The seismic stratigraphic record. *Bull. Geol. Soc. Am.* **122**, 830–842 (2010).

18. Huang, X. & Jokat, W. Middle Miocene to present sediment transport and deposits in the Southeastern Weddell Sea, Antarctica. *Glob. Planet. Change* (2016) doi:10.1016/j.gloplacha.2016.03.002.

19. Orejola, N. & Passchier, S. Sedimentology of lower Pliocene to upper Pleistocene diamictons from IODP site U1358, Wilkes Land margin, and implications for East Antarctic Ice Sheet dynamics. *Antarct. Sci.* **26**, 183–192 (2013).

20. Reinardy, B. T. I. *et al.* Repeated advance and retreat of the East Antarctic Ice Sheet on the continental shelf during the early Pliocene warm period. *Palaeogeogr. Palaeoclimatol. Palaeoecol.* **422**, 65–84 (2015).

21. Cook, C. P. *et al.* Dynamic behaviour of the East Antarctic ice sheet during Pliocene warmth. *Nat. Geosci.* **6**, 765–769 (2013).

22. Patterson, M. O. *et al.* Orbital forcing of the East Antarctic ice sheet during the Pliocene and Early Pleistocene. *Nat. Geosci.* **7**, 841–847 (2014).

23. Hansen, M. A., Passchier, S., Khim, B. K., Song, B. & Williams, T. Threshold behavior of a marine-based sector of the East Antarctic Ice Sheet in response to early Pliocene ocean warming. *Paleoceanography* **30**, 789–801 (2015).

24. Bertram, R. A. *et al.* Pliocene deglacial event timelines and the biogeochemical response offshore Wilkes Subglacial Basin, East Antarctica. *Earth Planet. Sci. Lett.* **494**, 109–116 (2018).

25. Escutia, C. *et al.* Cenozoic ice sheet history from East Antarctic Wilkes Land continental margin sediments. *Glob. Planet. Change* **45**, 51–81 (2005).

26. Williams, T. *et al.* Evidence for iceberg armadas from East Antarctica in the Southern Ocean during the late Miocene and early Pliocene. *Earth Planet. Sci. Lett.* **290**, 351–361 (2010).

27. Cook, C. P. *et al.* Sea surface temperature control on the distribution of far-traveled Southern Ocean ice-rafted detritus during the Pliocene. *Paleoceanography* **29**, 533–548 (2014).

28. Marschalek, J., Gasson, E., van de Flierdt, T., Hillenbrand, C.-D. & Siegert, M. A Path to Quantitative Interpretation of Antarctic Sediment Provenance Records. in *EGU General Assembly 2022* EGU22-1667 (2022). doi:10.5194/egusphere-egu22-1667.

29. Gulick, S. P. S. *et al.* Initiation and long-term instability of the East Antarctic Ice Sheet. *Nature* **552**, 225–229 (2017).

30. Cooper, A. K., Stagg, H. & Geist, E. L. Seismic stratigraphy and structure of Prydz Bay, Antarctica: Implications from Leg 119 drilling. in *Barron, J., et al, Proceedings of the Ocean Drilling Program, Scientific results, Volume 119* 5–25 (1991).

31. O'Brien, P. E. & Harris, P. T. Patterns of glacial erosion and deposition in Prydz Bay and the past behaviour of the Lambert Glacier. *Pap. Proc. - R. Soc. Tasmania* **130**, 79–85 (1996).

32. Passchier, S. *et al.* Pliocene-Pleistocene glaciomarine sedimentation in eastern Prydz Bay and development of the Prydz trough-mouth fan, ODP Sites 1166 and 1167, East Antarctica. *Mar. Geol.* **199**, 279–305 (2003).

33. Passchier, S. Linkages between East Antarctic Ice Sheet extent and Southern Ocean temperatures based on a Pliocene high - resolution record of ice - rafted debris off Prydz Bay, East Antarctica.

Paleoceanography **26**, (2011).

34. Whitehead, J. M., Harwood, D. M., McKelvey, B. C., Hambrey, M. J. & McMinn, A. Diatom biostratigraphy of the Cenozoic glaciomarine Pagodroma Group, northern Prince Charles Mountains East Antarctica. *Aust. J. Earth Sci.* **51**, 521–547 (2004).

35. Passchier, S. & Whitehead, J. M. Anomalous geochemical provenance and weathering history of Plio-Pleistocene glaciomarine fjord strata, Bardin Bluffs Formation, East Antarctica. *Sedimentology* **53**, 929–942 (2006).

36. Shakun, J. D. *et al.* Minimal East Antarctic Ice Sheet retreat onto land during the past eight million years. *Nature* **558**, 284–287 (2018).

37. Yamane, M. *et al.* Exposure age and ice-sheet model constraints on Pliocene East Antarctic ice sheet dynamics. *Nat. Commun.* **6**, 1–8 (2015).

38. Spector, P. *et al.* Miocene to Pleistocene glacial history of West Antarctica inferred from Nunatak geomorphology and cosmogenic-nuclide measurements on bedrock surfaces. *Am. J. Sci.* **320**, 637–676 (2020).

39. Halberstadt, A. R. W., Balco, G., Buchband, H. & Spector, P. Cosmogenic-nuclide data from Antarctic nunataks can constrain past ice sheet instabilities. *Cryosph.* **17**, 1623–1643 (2023).
